# A very rare case of priapism under aripiprazole in a patient followed for bipolar disorder: A CARE-compliant report

**DOI:** 10.1016/j.amsu.2021.01.015

**Published:** 2021-01-20

**Authors:** Salah-Eddine El Jabiry, Atif Mansour, Mohammed Barrimi, Bouchra Oneib, Fatima El Ghazouani

**Affiliations:** Department of Psychiatry, Mohammed VI University Hospital, Faculty of Medicine and Pharmacy, Mohammed I^st^ University, Oujda, Morocco

**Keywords:** Priapism, Aripiprazole, Antipsychotics, alpha1-adrenergic receptors, Case report

## Abstract

**Introduction and importance:**

Priapism is a urological emergency characterized by abnormally prolonged, painful and irreducible erection. It occurs without a sexual stimulation and habitually exceeds 6 h. About a half of iatrogenic priapisms are believed to be associated with antipsychotics. Until to date, very few cases of aripiprazole-associated priapism were reported.

**Case presentation:**

In this case report, we present the clinical findings of a 40-year-old patient that developed priapism after treatment with aripiprazole after his hospitalization for an episode of clinical mania following treatment discontinuation for bipolar I disorder. The management was successful and priapism was resolved spontaneously.

**Clinical discussion:**

Despite its low affinity to alpha-1 adrenergic receptors, aripiprazole may be associated with priapism. Several potential factors involved in the pathogenesis of this adverse event have been reported in the literature including history of priapism in a different class of neuroleptics and consumption of psychoactive drugs which are the principal factors found in our case.

**Conclusion:**

Priapism may occur even during treatment with antipsychotics that have a low affinity to alpha1-adrenergic receptors. All patients on antipsychotics should be informed about the risk of this rare but serious adverse event.

## Highlights

• Priapism is a urological emergency characterized by abnormal erection.
• Priapism can be associated with antipsychotics.
• Aripiprazole-associated priapism is very rare.
• Patients treated with aripiprazole should be informed about the risk of priapism.

## 1 Introduction

Priapism is a rare condition in which abnormally prolonged (>6 hours), painful, and irreducible erection without any sexual stimulations nor ejaculation can be observed [1]. Despite its rareness, it remains an extremely serious condition that requires appropriate management. Therefore, priapism is a medical and surgical emergency that may lead to severe sequelae, particularly, erectile dysfunction after fibrosis of the cavernous bodies [2]. Iatrogenic origins of priapism, especially associated with pharmacological treatments, are found implicated in 25–40% of the cases in which antipsychotics occupy 50% [3,4]. However, there are few published cases in the literature that reported aripiprazole-induced priapism. In this paper, we report a new case of this association in a patient treated for bipolar I disorder based on CARE-guidelines [5].

### 1.1 Presentation of case

Our patient is a 40 year-old male, married, father of a child, and working as a nurse. He has been followed for 18 years for bipolar I disorder diagnosed according to the Diagnostic and Statistical Manual of Mental Disorders V (DSM-V) criteria. He is a chronic user of tobacco at a rate of 30 packets/years and cannabis at a rate of 1 g per day. Cocaine was occasionally used. Moreover, he has a history of priapism induced by chlorpromazine during his hospitalization in 2016 for manic access. In that time, the management of his case required surgical intervention by puncture-washing of the cavernous bodies. After this adverse event, sedative neuroleptics were proscribed for him. Of note, our patient has no other personal or family history including psychiatric disorders. The disease history of our patient dates back to 2005, with the onset of clinical mania that required hospitalization and treatment with escalating doses of sodium valproate to 1500 mg/day. During follow up, the occurrence of several relapses of manic and depressive symptoms were noticed which required his hospitalization with free intervals between episodes. The patient was treated using single agents several including olanzapine (20 mg/day), risperidone (8 mg/day), and sertraline (50 mg/day). The treatments changes were proposed to the patient because of the marked adverse events reported principally sedation and priapism that needed his hospitalization in 2016.

In 2019, the patient was admitted to our psychiatric hospital for psychomotor excitement, tachypsychia with logorrhea, mood lability, ideas of grandeur and multiple projects, which were associated with insomnia and a tendency to hetero aggressiveness. According to DSM-V criteria, a mania in the context of bipolar I disorder following discontinuation of treatment for negative insight was retained as a diagnosis. Notably, there were no linguistic, cultural or financial issues during the management of our patient. Initially, our patient was treated with aripiprazole at a dose of 15 mg/day in combination with diazepam (30 mg/day). On day 10 of his hospitalization, the patient presented with a persistent and painful erection. He was referred to the urology department for management. A preliminary clinical assessment found no urological abnormalities. The diagnosis of priapism induced by aripiprazole was therefore retained. Fortunately, the erection resolved spontaneously, and the patient started benzodiazepines alone (diazepam 30 mg/day) with surveillance of his clinical and mental status. Subsequently, sodium valproate (1500 mg/day) combined with psychoeducation were proposed to the patient. The choice of this molecule was based on the good improvement and tolerance seen in the previous episodes as reported by the patient. The evolution was marked by the disappearance of manic symptoms, good tolerance, as well as non-recurrence of priapism.

## 2 Discussion

Despite the occurrence of aripiprazole-induced priapism is rare, its severity and difficult management should alert the attention of clinicians. Remarkably, a significant strength of our clinical case is the absence of any drug interactions that might justify the association of other molecules with priapism. In fact, our patient was treated with aripiprazole and diazepam only. Importantly, the recognition of this adverse event by the patient allowed us to intervene quickly. This is not always possible especially in patients with non-stabilized mental disorders. The selection of an appropriate treatment for the patient (sodium valproate) was guided by the nature of his psychiatric disorder (bipolar disorder type 1), the previous response to this treatment and the absence of affinity for alpha-1-adrenergic receptors. Therefore, this medication for this indication does not seem to increase the risk for developing priapism.

Several lines of evidence on the occurrence of priapism when using antipsychotics have supported the neuromuscular hypothesis that remained the most adopted worldwide. It suggests that antipsychotics-associated priapism depends on the ability of alpha-1-adrenergic receptors blockade of the cavernous bodies [6]. The current published literature reported several clinical cases of priapism associated with the two classes of antipsychotics including classical and atypical molecules [7]. In this perspective, haloperidol, chlorpromazine, levomepromazine and thioridazine are among the classical neuroleptics that have the greatest affinity for alpha-1 adrenergic receptors. Clozapine, quetiapine, risperidone and olanzapine are the atypical antipsychotics with high affinity for these receptors [[8], [9], [10]]. However, aripiprazole has the lowest affinity for this receptor among all atypical antipsychotics [11] (Table 1). Besides, despite this characteristic, cases of priapism induced by aripiprazole have been reported (Table 2). Two previous reports suggested an association between the dose of aripiprazole and priapism [12,13]. In 2006, Mago et al. discussed a case of recurrent priapism that was associated with the administration of aripiprazole [14]. In addition, priapism has been reported when aripiprazole was combined with oxcarbazepine and lithium [15]. Other authors reported a case of priapism with 10 mg of aripiprazole within a few hours of its first administration to patients with schizophrenia [16,17].

**Table 1:** Affinity of antipsychotics to alpha-1 adrenergic receptors.

Antipsychotics	Affinity
Amisulpride	**-**
Aripiprazole	**±**
Olanzapine	**++**
Clozapine	**+++**
Haloperidol	**+++**
Quetiapine	**+++**
Risperidone	**++++**
Chlorpromazine	**++++**

**Table 2:** Summary of published cases and reports on aripiprazole-associated priapism.

Author/year	Article type	Country of origin	Patient’ gender and age	Psychiatric disorder	Treatments and outcomes
Negin et al., 2005 [15]	Letter to the editor	USA	-Male −16	Pervasive developmental disorder and bipolar disorder	-Oxcarbazepine combined to lithium and aripiprazole -After two days of this treatment, two discrete episodes of prolonged penile erection were reported -No further prolonged erections were recorded after maintenance treatment with lithium and aripiprazole only
Mago et al., 2006 [14]	Letter to the editor	USA	-Male −47	Chronic paranoid schizophrenia	-Aripiprazole (dose not reported) -Recurrent priapism treated by cavernal irrigation and phenylephrine each time combined to pseudoephedrine -Lost to follow-up after discharge
Aguilar et al., 2009 [13]	Letter to the editor	Spain	-Male −23	Schizophrenic disorder	-Aripiprazole (20 mg/day) and dipotassium clorazepate. -Increase in the dose to 30 mg/day following the activation of the disease -Painful erection after two days of treatment for more than 24 hours -No similar episodes were noticed after urological intervention and dose reduction of aripiprazole to 20mg/day
Hsu et al., 2011 [12]	Letter to the editor	Taiwan	-Male −24	Psychotic disorder (not specified)	-Initial aripiprazole at a dose of 10mg/day with gradual increase to up to 25mg -Priapism was observed following this monotherapy -Switching to olanzapine (10 mg/day) was effective for priapism disappearance
Togul et al., 2012 [16]	Conference abstract	Turkey	-Male −30	Schizophrenia	-Aripiprazole at a dose of 10 mg/day. -The patient reported a painful erection 8 hours later. -Priapism disappeared after switching to olanzapine (20 mg/day).
Trivedi et al., 2016 [17]	Case report	India	-Male -An adolescent (age non specified)	Paranoid schizophrenia	-Aripiprazole 10 mg/day and lorazepam 2 mg. -Priapism after 7 hours treated successfully with blood aspiration with saline in combination to local adrenaline

Our patient case differs from the rare reports published mainly by the nature of the underlying psychiatric disease. On the other hand, other common points are present. The repeated occurrence of priapism under different antipsychotics could refer to a predisposition to this adverse event [18,19]. The use of psychoactive substances would also increase the risk of occurrence of priapism [20]. This was the case of our patient who is a chronic user of tobacco, cannabis and occasional cocaine. So far, the occurrence of priapism in our case does not appear to be dose related. In fact, our clinical case must attract the attention of practitioners to several key points. First, the systematic search for a history of priapism before administration of an antipsychotic should be performed because the literature raises cases of repeated priapism in these patients. Furthermore, a careful follow up of patients treated with antipsychotics especially those who have unstable psychiatric diseases should be implemented. And finally, the use of antipsychotics with weak or absent alpha1-adrenergic affinity should be recommended along with psychoeducation. During follow up, our patient was very satisfied with our management despite the stress experienced during his hospitalization for priapism.

## 3 Conclusion

Priapism is a serious urological emergency that requires a rapid and an adequate management. The factors predisposing to its occurrence after aripiprazole use or other antipsychotics need to be evaluated in well conducted robust studies. This will be essential to identify patients at risk to develop priapism. Patients under treatments using antipsychotics should be informed of the risk of this adverse event.

## Conflict of interest

None declared.

## Sources of funding

None.

## Ethical approval

N/a.

## Consent

Written informed consent was obtained from the patient for publication of this case report. A copy of the written consent is available for review by the Editor-in-Chief of this journal on request.

## Author contribution

Dr Salah-Eddine El Jabiry collected data and wrote the manuscript. Dr Atif Mansour participated in the patient management. Professors Mohammed Barrimi, Bouchra Oneib, and Fatima El Ghazouani supervised the article writing. All the authors approved the final version.

## Research registration

N/a.

## Guarantor

Dr Salah-Eddine El Jabiry.

## Provenance and peer review

Not commissioned, externally peer-reviewed.
