# Supplementary material for: A very rare case of priapism under aripiprazole in a patient followed for bipolar disorder: A CARE-compliant report
Source: Ann Med Surg (Lond). 2021 Jan 20;62:216–8. doi: 10.1016/j.amsu.2021.01.015 (PMC7843361; doi:10.1016/j.amsu.2021.01.015)
Supplement: Multimedia component 1 [file mmc1.docx]

**Title 1** The words “case report” should be in the title along with the area of focus . . . . . . . . . . . . . . . 1. . . . . . . . . . . . . . . . .

**Key Words 2** 2. to 5 key words that identify areas covered in this case report. . . . . . . . . . . . . . . . . . 56. . . . . . . . . . . . . . . . . . .

**Abstract**

**3a** Introduc ion. — . .W ha t is unique about this case? What does it add to the medical literature? . . . . . 36 , 39, 40 . . . . . . .

**3b** The main symptoms of the patient and the important clinical findings . . . .42, 43 . . . . . . . . . . . . . . . . . . . . . . . . . . . . .

**3c** The main diagnoses, therapeutics interventions, and outcomes . . . . . . . . . . . . . . . . . .43, 44. . . . . . . . . . . . . . . . . . . . . . . . .

**3d** Conclusion—What are the main “take-away” lessons from this case? . . . 52, 53. . . . . . . . . . . . . . . . . . . . . . . . . . . . . . . . . .

**Introduction**

**4** One or two paragraphs summarizing why this case is unique with references . . . . 77, 79, 82, 83. . . . . . . . . . . . . . . . . .

**Patient Information**

**5a** De-identified patient specific information . . . 89 . . . . . . . . . . . . . . . . . . . . . . . . . . . . . . . . . . . . . . . . . . . . . . . . . . . . . . .

**5b** Main concerns and symptoms of the patient . . . . . . . . . . . . . . 90. . . . . . . . . . . . . . . . . . . . . . . . . . . . . . . . . . . . . . . . .

**5c** Medical, family, and psychosocial history including relevant genetic information (also see timeline) . 90

**5d** Relevant past interventions and their outcomes . . . . . . . . 93, 94, 95. . . . . . . . . . . . . . . . . . . . . . . . . . . . . . . . . . . . . . . . . . .

**Clinical Findings**

**6** Describe the relevant physical examination (PE) and other significant clinical findings. . **. .** 115. . . . . . . . . .

**Timeline 7**

Important information from the patient’s history organized as a timeline . . . 98, 99, 100 ,101 , 102, 103, 104 . . . . . . . . . . . .

**Diagnostic**

**Assessment**

**8a** Diagnostic methods (such as PE, laboratory testing, imaging, surveys). . . . . . . . . . . . . 110 . . . . . . . . . . . . . . . .

**8b** Diagnostic challenges (such as access, financial, or cultural) . . . . . . . . 112, 113 . . . . . . . . . . . . . . . . . . . . . . . . . . . . . .

**8c** Diagnostic reasoning including other diagnoses considered . . . . . . . . . . . . . . . 114, 115, 116. . . . . . . . . . . . . . . . . . . . . . . . .

**8d** Prognostic characteristics (such as staging in oncology) where applicable . . . . . . . . . . . 118. . . . . . . . . . .

**Therapeutic**

**Intervention**

**9a** Types of intervention (such as pharmacologic, surgical, preventive, self-care) . . .119 . . . . . . . . . . . . . . . . . . . . . . . . . . . .

**9b** Administration of intervention (such as dosage, strength, duration) . . . . . . . . . . . . . 120, 121. . . . . . . . . . . . . . . . . . . . . . . .

**9c** Changes in intervention (with rationale) . . . . . . . . . . . . . . . . . . . . . . . 122 . . . . . . . . . . . . . . . . . . . . . . . . . . . . . . . . . . . . . . .

**Follow-up and**

**Outcomes**

**10a** Clinician and patient-assessed outcomes (when appropriate) . . . . . . . . 124. . . . . . . . .. . . . . . . . . . . . . . . . . . . . . . . . . . . .

**10b** Important follow-up diagnostic and other test results . . . . . . . . . . . . . . . 124. . . . . . . . . . . . . . . . . . . . . . . . . . . . . . . . . . . . .

**10c** Intervention adherence and tolerability (How was this assessed?) . . . . . . . . .124 . . . . . . . . . . . . . . . . . . . . . . . . . . . . . . . .

**10d** Adverse and unanticipated events . . . . . . . . . . . . . . . . . 124, 125 . . . . . . . . . . . . . . . . . . . . . . . . . . . . . . . . . . . . . . . . . . . . . . . . .

**Discussion**

**11a** Discussion of the strengths and limitations in your approach to this case . . 129, 130, 131, 132, 133, 134. . . . . . . . . . . . . . . . . . . . . . . . . . . . . . . . .

**11b** Discussion of the relevant medical literature . . . . . . . 142, 143, 144, 146, 147, 148, 149, 150, 151, 152, 153, 154, 155. 156, 157, 158…… 282, 294. . . . . . . . . . . . . . . . . . . . . . . . . . . . . . . . . . . . . . . . . . . . . . . .

**11c** The rationale for conclusions (including assessment of possible causes) . . . . 169, 170, 171. . . . . . . . . . . . . . . . . . . . . . . . .

**11d** The primary “take-away” lessons of this case report . . . . . . . . . 181, 182. . . . . . . . . . . . . . . . . .. . . . . . . . . . . . . . . . . . . . . . . .

**Patient Perspective 12** When appropriate the patient should share their perspective on the treatments they received . . .

175, 176, 177 . . . . . . . . . . . . . .

**Informed Consent 13** Did the patient give informed consent? Please provide if requested . . . . . . . . . . . 196. . . . .. . **Yes**
